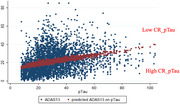# Impact of pTau‐Derived Cognitive Reserve on Alzheimer's Disease Progression

**DOI:** 10.1002/alz70857_106529

**Published:** 2025-12-24

**Authors:** Yeshin Kim, Yaakov Stern, Na‐Young Yeo, Hyemin Jang

**Affiliations:** ^1^ College of Medicine, Kangwon National University, Chuncheon, Gangwon‐do, Korea, Republic of (South); ^2^ Cognitive Neuroscience Division, Columbia University, New York, NY, USA; ^3^ Kangwon National University, Chuncheon, Korea, Republic of (South); ^4^ Seoul National University Hospital, Seoul National University College of Medicine, Jongno‐gu, Seoul, Korea, Republic of (South)

## Abstract

**Background:**

Alzheimer's disease (AD) follows a characteristic pathology where amyloid‐beta (Aβ) accumulation leads to tau hyperphosphorylation (pTau), neurodegeneration, and cognitive decline. While pTau is a key biomarker of AD progression, clinical outcomes vary among individuals with similar pathology. Cognitive Reserve (CR) may explain this variability by preserving cognitive function despite pathology. As AD diagnosis increasingly relies on biomarkers, understanding CR at the biomarker level is crucial. This study investigates the impact of CR based on pTau in AD progression, exploring whether higher CR is associated with a lower risk of disease progression.

**Method:**

We included amyloid‐positive 695 participants (132 CU, 368 MCI, 195 dementia) who underwent neuropsychological assessment, CSF Aβ1‐42, and CSF pTau analysis. The participants’ amyloid positivity was decided using CSF Aβ1‐42 level. ADAS13 was used for the cognitive function score. CR based on pTau was measured as the difference between the predicted and observed value of cognitive function based on CSF pTau level (CR_pTau). The participants were divided into two groups: a low‐CR_pTau group that showed a worse ADAS13 score than the predicted ADAS13 score on the pTau level and a high‐CR_pTau group that showed a better ADAS13 score than the predicted ADAS13 on pTau level. A mixed effect model was used to examine the difference in progression slope according to the CR_pTau group in each disease stage: CU, MCI, and dementia. Using survival analysis and cox proportional hazard model, we investigated the risk of progression of disease stage in two CR groups.

**Result:**

Mean age was 73.7 years, and 42.2% were female. The baseline proportion of dementia patients was higher in the low CR_pTau group compared to the high CR_pTau group. The high CR_pTau group showed slower cognitive decline compared to the low CR_pTau group across all disease stage (*p* <0.001). The high CR_pTau group showed 45% lower risk of disease stage progression to any stages compared to the low CR_pTau group (*p* <0.001). Progression of CDR‐SB was also slower in the high CR_pTau group and showed 28% lower risk of progression compared to the lower CR_pTau group (*p* <0.001)

**Conclusion:**

pTau‐derived CR might have impact on disease progression of AD especially.